# Precision Medicine 2.0: How Digital Health and AI Are Changing the Game

**DOI:** 10.3390/jpm13071057

**Published:** 2023-06-28

**Authors:** Daniele Giansanti

**Affiliations:** Centre TISP, ISS, 00161 Rome, Italy; daniele.giansanti@iss.it

In the era of rapid IT developments, the *health domain* is undergoing a considerable transformation [1]. The integration of *Digital Health* (DH) with *Artificial Intelligence* (AI) has paved the way for Precision Medicine 2.0, a groundbreaking approach that holds the promise of revolutionizing patient care [2,3,4]. The potential implications of this transformation are widespread, as it empowers healthcare professionals to deliver tailored treatments and improve patient outcomes. It is possible to identify the key contributions of this transformation for both AI and DH.

It can generally be stated that the *medico-biological elements of information* are treated by AI and DH with different roles and approaches. The first, AI, *mainly deals with the intelligent processing of this information, transforming it into decisions and therapeutic activations with the patient at the center* [5,6,7,8,9,10]; the second, DH, *takes care of its transmission, the taking it (also from the patient through sensors), and transporting/delivering information to the decision-making and activation nodes of the healthcare system, and also by developing innovative devices* [11,12,13,14,15,16,17,18].

AI makes a significant contribution, enabling the processing of large volumes of complex data and producing tailored information about a patient with a predictive capacity for the improvement and fine-tuning of therapeutic path in all phases.

AI enables [5,6,7,8,9,10]:

*-Data analysis:* Thanks to AI, it is possible to analyze large datasets (e.g.; BIG-DATA) and to extract calibrated intervention models that would otherwise be impossible, through, for example, the identification of more accurate prognostic, diagnostic, and predictive markers for specific diseases.

*-AI-assisted diagnosis:* AI has the potential to provide important support to instrumental diagnosis with specific algorithms, such as in the case of medical diagnostics, in digital pathology, digital radiology, digital dermatology.

*-Personalization of the treatment, monitoring, and management of disease*: AI, through the analysis of clinical and molecular data combined with information obtained from large external electronic data archives, can enable tuning and personalized treatment optimization; the elaboration of physiological parameters derived from wearable devices enables monitoring, and if necessary, adaptation of a patient’s care.

*-Predictive medicine:* AI can develop an interoperable data connection from the patient to the healthcare system and vice versa, by applying continuously updated algorithms, which provide and uses distributed *medical knowledge*, and can be used for predictive purposes of pathologies based on risk analysis.

*-Production of medical knowledge:* In all the activities described, AI contributes to the research and development of clinical and medical practice on various scales.

DH plays a fundamental role in precision medicine, enabling the interconnection of medico-biological data and the creation of technological solutions that support the personalization of care that may use AI and/or other decisional approaches based on algorithms [11,12,13,14,15,16,17,18]. 

DH, for example, enables:

*-Personalized data collection:* DH enables the collection of detailed patient data. Wearable devices allow, by means of specific sensors, the monitoring of physiological parameters, lifestyles, diets, and other useful information. These data can be integrated with further information from other databases.

*-Remote Monitoring:* Digital technologies, such as wearable devices, also enable continuous monitoring of the patient to modify the therapy and/or activate emergency actions when needed.

*-Data Sharing:* DH enables data sharing while respecting cybersecurity. The secure integration of data from many sources, including electronic patient records, diagnostic images, laboratory data, and genomic data, is therefore possible. 

*-Clinical Decision Support:* DH can provide the HW/SW base for clinical decision support systems using AI or tools for data analytics. 

*-Telemedicine*: Telemedicine enables the remote delivery of healthcare services. Thanks to advanced DH solutions (also using AI), telemedicine can be increasingly tailored to an individual patient.

Targeted searches on PubMed suggest the scale of growth in the volume of studies in this area.

Regarding the studies in precision medicine, searches for the keywords reported in Box 1 *position* 1 highlighted 20,160 studies starting from 1979. Of these studies, 11,646 (57.7%) had been carried out starting from 1 January 2020. In all, there were 8142 reviews (systematic and non-systematic).

Regarding the studies on precision medicine focused on DH, searches for the keywords reported in Box 1 *position 2* highlighted 94 studies starting from 2013. Of these studies, 65 (69.9%) had been carried out from 1 January 2020. In all, there were 37 reviews (systematic and not).

Regarding the studies on precision medicine focused on AI, searches for the keywords reported in Box 1 *position 3* highlighted 796 studies starting from 2015. Of these studies, 670 (84, 2%) had been carried out from 1 January 2020. In all, there were 438 reviews (systematic and non-systematic).

This brief overview highlights how, in these sectors, there has been an acceleration of scientific production and interest during the COVID-19 pandemic period; interest in studies on AI and DH is more recent (the 2000s); the comparison between AI and DH indicates greater interest in AI, i.e., a stronger interest in an intelligent IT approach than for the architecture of the information flow; and there is a good proportion of reviews for both AI and DH, indicating good progress in the stabilization of topics of scientific interest (Figure 1).

Box 1 **Composite key used for the searches in PubMed.**

                        (precision medicine[Title/Abstract])

                (precision medicine[Title/Abstract]) AND (digital health[Title/Abstract])

              (precision medicine[Title/Abstract]) AND (artificial intelligence[Title/Abstract])

Precision medicine has an older history than expected; the first studies date back to 1979. Its meaning has evolved [1,19] together with the expectations that scholars have gradually placed on it. Today, it could change healthcare both as we know it and how we evaluate it [20,21].

Emerging technologies, such as AI and DH (both individually and as a whole) are making an important contribution to the developments of this discipline. A real integration with the *health domain* will have to respect all the domains of action, from regulatory to ethical spheres.

There is an urgent need for discussion in this area to exchange and share universal experiences, both on opportunities and on problems and even failures. With this in mind, the Special Issue, entitled “Transforming Precision Medicine: The Intersection of Digital Health and AI” [22] was launched.

## Conclusions

The COVID-19 pandemic has led to a considerable acceleration in research and development on the applications of AI and DH in the precision medicine.

Scholars, experts, professionals, and stakeholders in the health domain are working both on the developments and integration on multiple domains. There is an increasing need for studies focused on AI and DH in clinical imaging, as well as synergistic initiatives such as collections or Special Issues which touch on both successes and failures, as well as opportunities and bottlenecks.

## Figures and Tables

**Figure 1:**
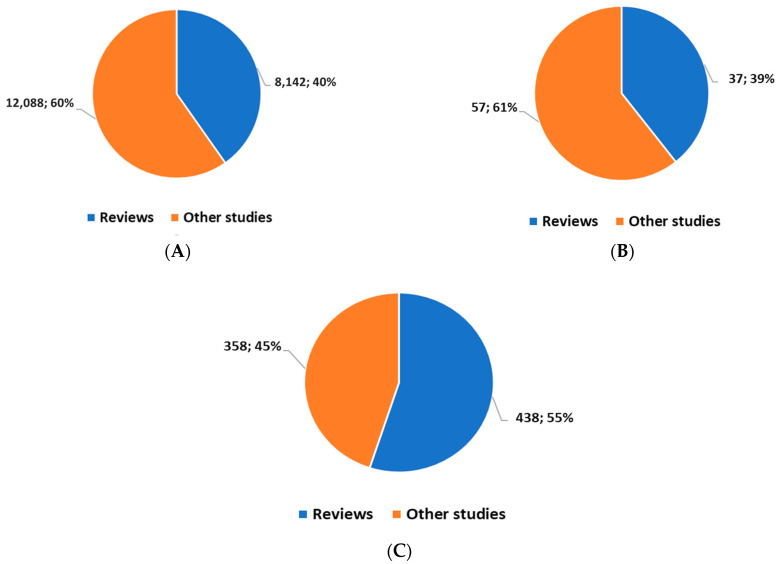
Volume of publications for the field of precision medicine (**A**); for the field of precision medicine and DH (**B**); and for the field of precision medicine and AI (**C**).
